# Oxidative Stress and Autophagy as Key Targets in Melanoma Cell Fate

**DOI:** 10.3390/cancers13225791

**Published:** 2021-11-18

**Authors:** Elisabetta Catalani, Matteo Giovarelli, Silvia Zecchini, Cristiana Perrotta, Davide Cervia

**Affiliations:** 1 Department for Innovation in Biological, Agro-Food and Forest Systems (DIBAF), Università degli Studi della Tuscia, Largo dell’Università snc, 01100 Viterbo, Italy; ecatalani@unitus.it; 2 Department of Biomedical and Clinical Sciences “Luigi Sacco” (DIBIC), Università degli Studi di Milano, Via G.B. Grassi 74, 20157 Milano, Italy; matteo.giovarelli@unimi.it (M.G.); silvia.zecchini@unimi.it (S.Z.)

**Keywords:** reactive oxygen and nitrogen species, autophagosomes, cell death and survival, anti and pro-oxidants, autophagy modulators, skin cancer

## Abstract

**Simple Summary:**

Oxidative stress occurs when the balance between oxidants and antioxidant factors is disrupted in biological systems. This is a key field of study likely involved in many diseases, including skin cancers. In this respect, the oxidative stress phenotype in melanoma cells is well established as well as the connections with autophagy, an evolutionarily conserved catabolic process in eukaryotes that has an essential role in maintaining melanoma cellular homeostasis in response to intracellular stress. Herein, we present the evidence for drugs and treatments that are able to modulate melanoma cell fate via oxidative stress and autophagy, since the development of successful therapies in skin cancer depends on a more complete understanding of the cross-talk between these events and their pharmacological targeting.

**Abstract:**

Melanoma originates from the malignant transformation of melanocytes and is one of the most aggressive forms of cancer. The recent approval of several drugs has increased the chance of survival although a significant subset of patients with metastatic melanoma do not show a long-lasting response to these treatments. The complex cross-talk between oxidative stress and the catabolic process autophagy seems to play a central role in all aspects of melanoma pathophysiology, from initiation to progression and metastasis, including drug resistance. However, determining the fine role of autophagy in cancer death and in response to redox disruption is still a fundamental challenge in order to advance both basic and translational aspects of this field. In order to summarize the interactions among reactive oxygen and nitrogen species, autophagy machinery and proliferation/growth/death/apoptosis/survival, we provide here a narrative review of the preclinical evidence for drugs/treatments that modulate oxidative stress and autophagy in melanoma cells. The significance and the potential for pharmacological targeting (also through multiple and combination approaches) of these two different events, which can contribute independently or simultaneously to the fate of melanoma, may help to define new processes and their interconnections underlying skin cancer biology and unravel new reliable approaches.

## 1. Introduction

Melanoma is one of the most aggressive forms of cancer, with a global incidence rate of 3–5 per 100,000 patients, which is continuously rising, and a very high mortality rate (65% of skin cancer-related deaths) [1]. Melanoma presents a variety of subtypes, differing in terms of anatomical localization, growth patterns and genetic aberrations [2]. Thus, in 2018 the World Health Organization revised the classification of melanoma based on clinical, histologic, epidemiologic and genomic characteristics [3].

When the term melanoma is used, it is generally referring to cutaneous melanoma, because the vast majority of melanomas are of the skin. Cutaneous melanoma originates from the malignant transformation of cells of the neural-crest-derived melanocytic lineage, primarily melanocytes, cells of the basal layer of the epidermis that are responsible for producing the pigment known as melanin [4,5,6]. The involvement of other cells, such as melanocyte precursor and dermal stem cells, in the origin of melanoma has been proposed and is still under investigation [7,8,9,10]. Nevi such as common acquired nevi, dysplastic nevi and congenital nevi are considered precursor lesions of melanoma, although several melanomas may arise from clinically undetectable precursor lesions. According to the spread of the tumour, melanoma can be classified into five distinct stages (from stage 0 to IV) [11]; stage 0 is known as melanoma in situ while stage IV melanoma is defined as metastatic melanoma. Initially, melanoma is characterised by a radial growth phase (RGP), in which tumour cells are confined to the epidermis and, although invasive, have little or no metastatic competence. Then, melanoma cells acquire the ability to invade, survive and proliferate in the papillary dermis such that tumour enters the vertical growth phase (VGP). Once cells have spread to distant organs, the disease is known as metastatic melanoma [11]. It should be noted that not all forms of melanoma follow the canonical transition from RGP to VGP, as, for instance, nodular melanoma is a lesion lacking a recognizable RGP but with a rapid VGP and therefore with a high potential to generate metastasis from its first diagnosis [12].

In melanoma, as in other tumours, progression has been clearly related to the progressive acquisition of genetic alterations and each stage has different molecular drivers [13,14,15,16,17]. The prevalent mutations in melanoma are in genes encoding for effectors of the Ras pathway such as BRAF or NRAS [18,19]. However, mutations in BRAF have been found in up to 80% of benign nevi that rarely further progress to melanoma, thus indicating that BRAF alteration alone is not sufficient for melanoma development [19,20]. Mutations of NRAS, which are associated with an aggressive clinical course and a poor prognosis, occur in more than 20% of cutaneous melanoma patients [21]. Loss of function alterations in the cyclin dependent kinase inhibitor 2A (CDKN2A) locus are present in 25–35% of melanoma with a higher frequency in patients with a strong family history of melanoma, in comparison to those with sporadic melanoma, and are involved in the genesis of malignant cutaneous melanoma by generally occurring at the stage of dysplastic nevus [2,16,21,22]. CDKN2A encodes two tumour suppressor genes, CDKN2A/p16 and p14ARF, controlling cell cycle progression [23]. The progression into the stage of RGP is often related to the acquisition of mutations (40–50% of melanomas) in the promoter of the TERT gene, that encodes for telomerase reverse transcriptase and drives melanoma cell immortality [21,24,25]. Finally, for the entering of melanoma in the VGP, genetic defects are required in molecular players of cell survival and apoptosis such as phosphatase and tensin homolog (PTEN), occurring in 10% of melanomas, and tumour protein 53 (TP53), found to be mutated in approximately 15% of melanomas [2,16,21,26]. Other mutated genes found in melanomas belong to pathways fundamental for the progression of the metastatic disease, for instance mitogen-activated protein kinase (MAPK), phosphoinositide 3-kinase (PI3K), WNT, receptor tyrosine-protein kinase KIT and melanocyte inducing transcription factor (MITF) [27,28].

In the last years, different effective substances proposed for melanoma therapy have been shown to act primarily through mechanisms involving “oxidative stress”, an imbalance between oxidants and antioxidant factors which can damage biological systems [29], and the catabolic process “autophagy” [30,31]. However, determining the fine role of autophagy in melanoma cell death and in response to oxidative stress is still a fundamental challenge in order to advance both basic and translational aspects of this field. We present here a narrative review of this with the objective to highlight the landscape of potential pharmacological approaches as a modality for skin cancer treatments.

### 1.1. Oxidative Stress

Oxidative stress is considered an important translational field at the root of many diseases [32,33]. The theory of illness based on oxidative stress relies on the role of molecular oxygen (O_2_) in the cells. Indeed, the metabolism of O_2_ generates reactive oxygen species (ROS), for instance hydroxyl radical (•OH), hydrogen peroxide (H_2_O_2_), superoxide radical (•O_2_^−^), ozone and singlet oxygen. Of note, the reaction of nitric oxide (•NO) and superoxide produces another short-lived oxidant species, the peroxynitrite (ONOO^−^) anion, giving rise to the family of chemically reactive molecules containing nitrogen (reactive nitrogen species, RNS), causing nitrosative stress. These two classes of oxygen-containing species are indicated collectively as RONS, which mainly refers to hydrogen peroxide, superoxide, nitric oxide and peroxynitrite, and are responsible for oxidative stress [29,33,34]. Indeed, RONS react with several macromolecules, including structural proteins, enzymes, nucleic acids and membrane lipids, and damage the biochemical homeostasis of the cells (aberrant cell function and death) under certain circumstances.

In order to prevent cell/tissue damage, the limitation of •OH production is effective although its direct scavenging is impractical. For this reason, multiple and redundant enzymatic and non-enzymatic antioxidant defence systems exist to counteract RONS formation, among them superoxide dismutase, catalase, peroxidases, and endogenous (for instance, vitamins and glutathione) or exogenous low-molecular-weight molecules. The generation/removal of •OH, H_2_O_2_, •O_2_^−^, and •NO within levels that do not permit a significant formation of ONOO^−^ and •OH determines the effectiveness of these antioxidant systems [29]. It is important to specify that the predominant antioxidant effect inside cells relies on the antioxidant enzymes since they react much more rapidly with oxidants than small molecules do. In contrast, extracellular fluids which lack antioxidant enzymes and •O_2_^−^ or H_2_O_2_ can be efficiently prevented by small scavengers.

**Figure 1:**
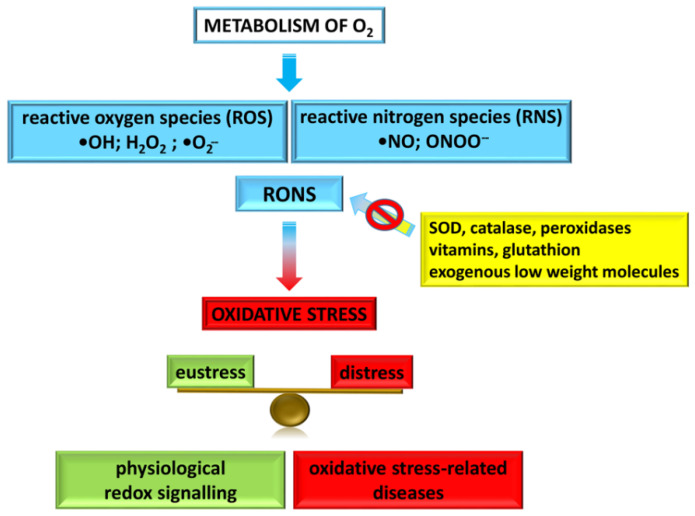
Schematic picture of the modern concept of oxidative stress.

Oxidative stress is not always detrimental but may have important physiological actions (commonly termed “redox signalling”). Thus, the concept of oxidative stress in pathology has evolved over time into redox signalling in physiology [29,35,36] (Figure 1). Indeed, for maintaining normal physiology in a process defined “adaptive homeostasis” [37] a balance is needed between oxidants and reductants [29,33], including nicotinamide adenine dinucleotide phosphate (NADPH), which is the ultimate donor of reductive power for many ROS-detoxifying enzymes. In this respect, disruption of redox homeostasis can be involved in oxidative stress-related diseases; in fact, RONS can dysregulate the same pathways as redox signalling under physiological conditions, causing nonspecific damage [35,36].

### 1.2. Autophagy

Autophagy is an evolutionarily conserved, lysosome-dependent and gene-regulated catabolic process in eukaryotes, whereby cytoplasmic components, for instance damaged organelles, protein aggregates and lipid droplets, are degraded for recycling [30]. Autophagy has various connections to human disorders, as it has an essential role in maintaining cellular homeostasis in response to intracellular stress and its functions are important for bioenergetics homeostasis and tissue/organism development. Of note, maintaining the delicate balance between the survival and the death of a cell is a key role for the process of autophagy, which has been studied profoundly in last decades [38]. In this respect, many efforts have been made to identify the autophagic machines, the functional targets and the pathophysiological roles of autophagy in different diseases, including neurodegenerative disorders, retinal defects, metabolic impairments and muscle-related pathologies [39,40,41,42,43,44,45,46,47,48,49,50,51,52,53,54,55].

It is well established that autophagy can inhibit the occurrence of tumours and prevent normal cells from becoming cancerous [40,51,56,57,58,59]. On the other hand, the process of autophagy may help tumour cell survival once carcinogenesis has occurred and, consequently, contributes to tumour progression. Additionally, autophagy has a role in the modulation of the cancer response to therapy, leading to radiotherapy and chemotherapy resistance or decreased susceptibility to antitumour drugs. In this regard, more research concerning the molecular machinery of autophagy molecular and potential autophagy-related targets needs to be conducted in cancer treatment and prevention.

## 2. Oxidative Stress-Autophagy-Melanoma in Brief and Review Criteria

A wide range of pathologies have been associated with oxidative stress/redox state disruption as the primary cause or as the secondary contributor to disease progression and much effort has been applied for the translation of antioxidant therapy into clinical application [33]. However, although multiple antioxidant strategies have been proposed (with some of them undergoing promising clinical trials) most of the approaches using general antioxidant therapy have failed. As previously suggested, the focus on specific oxygen-toxifying pathways rather than on non-specific RONS scavengers would be the best potential pharmaceutical intervention [60]. In melanoma cells, the existence of a redox imbalance, or oxidative stress phenotype, is well established [61] and plays a central role in all aspects of melanoma pathophysiology, from initiation to progression and metastasis, including drug resistance [62]. Overall, an elevated oxidative status has been associated with melanoma. In this respect, the experimental and potential clinical use of antioxidants in the pathophysiology of malignant melanoma has been recently summarised [63]. Briefly, counteracting oxidative stress seems to be a double-edged sword: it may induce melanoma cells to behave less aggressively or even undergo apoptosis; on the other hand, cell survival may be improved by antioxidants thus resulting in metastasis promotion and resistance to therapeutic regimes. For instance, a model of redox adaptation has been recently proposed regarding BRAF inhibitors resistance. Long-term treatment with BRAF inhibitors such as vemurafenib triggers the selection of resistant melanoma cells that, as a consequence of a mitochondrial respiration phenotype, exhibit increased ROS production and increased redox response [62].

Similarly, autophagy mechanisms are key cellular events as contributors to melanoma growth and tumour maintenance since they regulate melanoma cell death/survival, proliferation, apoptosis and chemotherapeutic effects [64,65]. Although autophagy induction may protect melanocytes and play a preventive role in early-stage melanoma formation, overall it seems that autophagy is activated mostly during the advanced stage of melanoma. Indeed, the analysis of primary melanoma in comparison to benign nevi showed a reduced expression of the proautophagic proteins Atg5, beclin-1, microtubule-associated protein 1A/1B-light chain 3 (LC3A and LC3B); moreover, the expression levels of p62, a ubiquitin-binding scaffold protein also called sequestosome-1, and activating molecule in Beclin 1-regulated autophagy protein 1 (AMBRA1), have been proposed as prognostic biomarkers of early-stage melanoma [66,67,68]. It has also been demonstrated that LC3 is involved in the progression and metastasis of highly pigmented melanoma and that high levels of LC3B correlate with metastasis, low therapeutic response and short overall survival in melanoma patients [68]. This is in line with results from studies suggesting that autophagy plays a cytoprotective role during cancer progression and chemotherapy resistance [69]. Interestingly, resistance to BRAF inhibitors dabrafenib and vemurafenib has been reported to likely be associated with the induction of autophagy. Furthermore, an increase of autophagy levels was found in melanoma biopsies from patients treated with either a BRAF inhibitor alone or in combination with a MEK inhibitor in comparison to the levels measured before initiating treatment; this upregulation of autophagy was further associated with a lower progression-free survival time [70,71]. However, autophagy stimulation can also be considered as a therapeutic approach when autophagic-induced cell death is needed as an alternative strategy in apoptosis-resistant melanomas. The signals modulating autophagy and the mechanisms by which autophagy modulates melanoma cell proliferation deserve to be evaluated in depth in order to find novel specific autophagy inhibitors and activators for skin cancers [64,65,72].

There is a general agreement that a complex cross-talk between RONS and autophagy exists [73,74]. Indeed, RONS modulates autophagy (through several different pathways) and autophagy, in turn, may change RONS levels in a feedback interaction which determines cell fate. Autophagy could be thus a key cellular mechanism regulating the occurrence of oxidative stress, by engulfing and degrading oxidized substances, or a destructive process [73,74]. The evidence for drugs which are able to modulate melanoma cell fate via oxidative stress and autophagy has evolved significantly. In order to summarize the interactions among RONS, autophagy machinery, and cell death/survival in melanomas, Pubmed searches were performed in July 2021. Articles containing the following keywords were considered for inclusion: oxidative stress (or ROS/RONS) AND autophagy AND melanoma. Relevant articles were also identified from a manual search of reference lists within those included. The abstracts of identified articles were screened and classified for inclusion or exclusion in the review. To be included, the article must have described original data on the effect of oxidative stress/autophagy agents or treatments in melanoma, and been published in a peer-reviewed journal and written in English. In particular, for each of the included studies we extracted: (i) the year of publication, (ii) the melanoma model (in vitro and in vivo) that was used, (iii) the compound/physical treatment/pharmacological approach (for instance, combinations) administered, (iv) their mechanisms of action/intracellular pathways (when appropriate), and (v) their effects on redox state, autophagy mechanisms and cell death/survival of melanoma cells.

## 3. Melanoma Cell Death and Survival: Simultaneous Regulation of Oxidative Stress System and Autophagy Machinery

The crucial role of autophagy and RONS in melanoma pathophysiology was highlighted using a BRAF^V600E^ mutant, PTEN tumour suppressor gene-null, Atg7-deficient mouse model of melanoma [75]. The mutants exhibited extended survival, accumulation of autophagic substrates p62 and LC3, increased oxidative stress and senescence. In addition, B16 cells were shown to be highly susceptible to oxygen partial pressure, since short-term and long-term hypoxia/reoxygenation treatment increased ROS production, apoptosis and autophagy [76].

One particular challenge to understand RONS–autophagy dynamics and the mechanistic relationship between RONS and autophagy in melanoma is in recognizing the biological significance and the potential for concomitant pharmacological targeting (also through multiple and combination approaches) of these two processes. RONS and autophagy, indeed, can contribute independently or simultaneously to the fate of melanoma cells (proliferation/growth/death/apoptosis/survival). Our search and screening strategy led to the following results, suggesting the relevance of this approach in melanoma studies.

### 3.1. Chemotherapeutics Agents and Approved Synthetic Drugs

Three classes of cytotoxic chemotherapy have been used in the treatment of melanoma: alkylating agents (dacarbazine, temozolomide), platinum compounds (cis-platinum, carboplatin) and mitotic inhibitors (paclitaxel) [77]. While no references were found in our search on a role for temozolomide and paclitaxel in the RONS–autophagy cross-talk, an article of Bedia and coworkers demonstrated that dacarbazine triggers the production of ROS and ensuing cell death with autophagic features via ceramide accumulation [78].

The well-known chemotherapeutic agent cisplatin was repurposed in association with fasting cycles as a clinical approach to treat melanoma cancer. Cisplatin was used in combination with the synthetic glucose analogue 2-Deoxy-D-glucose (2DG), known to block cell growth because of energy failure [79]. 2DG activity was tested in combination with pro-apoptotic agents other than cisplatin, including temozolomide, pyrimethamine, a pro-apoptotic antifolate drug and the kinase inhibitor staurosporine. 2DG alone promoted protective autophagy, reduced ROS levels and increased mitochondrial membrane potential in 8863 (MeWo) and Mel-501 cells, but did not induce a significant rate of apoptosis. Interestingly, 2DG increased apoptosis and mitochondria depolarization when used in combination with cisplatin or staurosporine, but not with temozolomide. On the contrary, 2DG in combination with pyrimethamine promoted cell survival by enhancing autophagy and mitochondria hyperpolarization, but decreased ROS level. Combined treatment with cisplatin and nutrient deprivation resulted in higher and earlier cell death induction in wild type and oncogenic BRAF^V600E^ SK-Mel 28 cells [80]. Cell death occurred mainly by apoptosis and ROS increase but without involvement of endoplasmic reticulum (ER) stress and autophagy. The combined effect with nutrient deprivation was tested even for the autophagy inhibitor chloroquine, used as an antimalaric and anti-inflammatory drug [81]. In B16 cells, chloroquine triggered lysosomal accumulation and oxidative stress, resulting in mitochondrial depolarization and apoptotic/necrotic cell death. In this respect, chloroquine treatment of cells resistant to cisplatin, characterised also by an alteration of oxidative metabolism, led to the re-sensitization to the chemotherapeutic drug through the modulation of mammalian target of rapamycin (mTOR)-mediated autophagy [82,83]. A similar scenario was observed in calorie-restricted mice in which chloroquine reduced melanoma growth and necrosis of tumour cells. Since tumour cells are sensitive to arginine deprivation, recombinant human arginase (rhArg) was tested on malignant melanoma. Results in A375 cells showed that rhArg induces autophagy with the involvement of mitochondrial potential loss, apoptosis, ROS generation and Akt (also known as protein kinase B or PKB)/mTOR signalling pathway; of note, ROS was suggested to play an important role in rhArg-induced cell growth inhibition and autophagy [84]. Similar results were achieved after combined therapy with the synthetic drug imiquimod with γ-ionizing radiation that induced cell death via autophagy activation in B16-F10 and B16-F1 cells [85]. Autophagy machinery appeared speeded up by ROS-mediated MAPK and nuclear factor kappa-light-chain-enhancer of activated B cells (NF-κB) signalling pathway. Of note, reduced tumour growth, metastasis and enhanced anticancer immunity were observed in B16 tumour-bearing mice.

The causal involvement of autophagy in melanoma cell death was confirmed using bafilomycin A1, chloroquine, and ammonium chloride alone. These lysosomal autophagy inhibitors induced caspase-mediated apoptosis of B16-F0 cells in parallel with high levels of oxidative stress and autophagy-independent mitochondrial depolarization [86]. In another study on B16 cells, metformin (N,N-dimethylbiguanide), an antidiabetic drug used for the treatment of type 2 diabetes, induced cell cycle arrest, oxidative stress, mitochondrial membrane depolarization and then apoptosis [87]. In addition, metformin-activated autophagy reduced tumour size in an in vivo melanoma model, and, of note, the cytotoxicity of cisplatin is improved by metformin while it is antagonised in other tumour cell lines via a pro-survival Akt signalling [88]. Using multiple melanoma cells, lopinavir, an anti-HIV protease inhibitor, and its derivative lopinavir-NO were shown to induce morphological changes, ROS production and slight apoptotic features, but lopinavir-NO exhibited stronger anticancer action than lopinavir both in vitro and in vivo [89]. Of note, autophagosomes were detected only in B16 cells, indicating a cell-line-specific treatment response, although autophagy as mediator of the anticancer mechanism of the compounds was excluded. However, activated autophagy that in turn can stimulate apoptosis was observed in A375, HT144 and Hs294T cells treated with the H1 histamine receptor antagonist terfenadine, which may increase ROS depending on culture condition [90]. Accordingly, in A375 and BLM cell lines, both autophagy and apoptosis were induced by the proteasome inhibitor bortezomib through the involvement, at least in part, of the ROS-mitochondrial dysregulation-associated pathways [91]. Mitochondrial dysfunction was also induced by treatment with the antimicrobial triclosan in A375 cells [92]. Triclosan caused cytotoxicity and cell death acting on ROS signalling and promoting autophagy. In this line, the combination of neratinib, an irreversible inhibitor of the tyrosine kinase family members ErbB1/2/4, was tested with the histone deacetylase inhibitor entinostat on uveal melanoma cells [93]. Neratinib-entinostat induced tumour cell death by multiple ways of action, including ROS-dependent pathways, causing mitochondrial dysfunction and increased autophagy.

Autophagy was found to be a crucial event of the antiparasitic drug ivermectin in SK-Mel 28 cells. Indeed, ivermectin enhanced autophagy via ROS signalling pathways and caused cell death by apoptosis [94]. Interestingly, pharmacological or genetic abrogation of autophagy increased the ivermectin-induced apoptosis. Besides, inhibition of autophagy is one of the toxic ways of action of the proton pump inhibitor esomeprazole, which exerted ROS-dependent cell death in Me30966, Mel501 and WM793 cell lines [95]. It caused melanoma cell death via caspase-dependent pathway, also reducing autophagic flux. To note, autophagocytic degradation of melanosomes and ROS formation were induced by treatment with monobenzone in M0508A melanocytes and melanoma cells (92.2, 136.2, and WBO). Monobenzone is a vitiligo-inducing compound that suppresses cellular pigment synthesis, induced dendritic cells activation, and stimulated cytotoxic melanoma-reactive T cells that, in turn, eradicated melanoma in vivo [96].

### 3.2. Test Synthetic Compounds

The use of nanoparticles for drug delivery could represent an alternative strategy in melanoma therapies. Recently, Ag@ZnO nanoparticles under ultraviolet (UV) exposure were shown to increase ROS production in A375 cells leading to cytotoxicity, increased autophagic turnover and cell death via apoptosis [97]. In A375 cells, photo-activated nitrogen-doped titanium dioxide nanoparticles (N-TiO_2_ NPs) induced an increase in ROS production that led to necroptosis and the blockade of autophagy attributable to a variation in lysosomes acidity [98]. On the contrary, in dark conditions, N-TiO_2_ NPs enhanced autophagic flux as a pro-survival strategy. Similarly, N-P-doped carbon dots displayed anticancer properties against B16-F10 cells since they enhanced cytotoxicity and promoted apoptosis, oxidative stress and autophagy [99]. In another set of experiments, the anticancer potential of cisplatin loaded on the drug delivery system Santa Barbara amorphous 15 (SBA-15|CP) was evaluated on B16-F1 cell line and in the syngeneic melanoma mouse model [100]. While SBA-15 alone did not produce any effect on B16-F1 cells, SBA-15|CP showed a similar high cytotoxic activity of free cisplatin causing apoptosis and inhibition of cell proliferation. In addition, weak autophagy flux and no production of ROS was observed. However, a high level of NO was detected. Remarkably, unlike naked cisplatin, in in vivo experiments, SBA-15|CP diminished tumour volume and side effects.

Still, some substances exert positive effects in vitro but not in vivo, for instance the heat shock protein 90 (hsp90) inhibitor NVP-AUY922 [101], which inhibited A375 cell growth but failed to counteract melanoma development in vivo. However, an increase in cell death and a slowing down of tumour growth were observed using NVP-AUY922 in combination with 2-phenylethynesulphonamide (PFT-μ), an inhibitor of hsp70 and autophagy. It was proposed that the oxidative stress caused by PFT-μ promotes NVP-AUY922-induced cytotoxic effects. In addition, bis(phenylidenebenzeneamine)-1-disulfide derivative compound 2 induced ROS generation and displayed antiproliferative effects in A2058, RPMI7951 and B16 cell lines, and in vivo mouse melanoma models, although its role on apoptosis and autophagy was not defined [102]. Of interest, in vivo inhibition of tumour growth was observed after BAY 87-2243 treatment, a potent inhibitor of mitochondrial complex I [103]. In addition, it induced cell death, increased cellular ROS levels, enhanced lipid peroxidation and decreased glutathione levels in G361 and SK-Mel 28 cells, stimulating autophagosome formation and mitophagy. To describe BAY 87-2243 action, a cascade of events was proposed, which comprises complex I inhibition, autophagosome formation, mitophagy, ROS increase and activation of combined necroptotic/ferroptotic cell death. Similarly, the synthetic peptide AC-1001-H3 (based on mouse antibody AC-1001 H3 CDR peptide) exerted antimetastatic action on a syngeneic model with B16-F10-Nex2 cells, as well as cytotoxic effects on B16-F10-Nex2 and A2058 cells, apoptosis, ROS production and enhanced autophagy [104].

Another example came from the synthetic antitumour compound 1,4-naphthoquinone derivative CB533, which induced AKT-dependent autophagy in A375 cells and mimicked ROS-induced stress signalling without causing ROS production and apoptosis [105]. Recently, a semisynthetic p-quinol, protoapigenone 1′-*O*-butyl ether (PABut), was tested as an antimelanoma agent in parallel with its natural congener protoapigenone (PA), a derivative of apigenin [106]. Both compounds showed cytotoxicity toward A375 cells and tumour selectivity. Indeed, PABut and PA increased apoptosis at the early and the late stage, respectively. Moreover, PABut was more effective than PA in enhancing autophagy although PABut and PA similarly stimulated ROS production, which also promoted senescence. On B16 cells, octahedral platinum(IV) complexes (satraplatin analogues) exerted anticancer activity through a non-apoptotic cell death associated with cell membrane damage. Of note, octahedral platinum(IV) complexes induced oxidative stress and necrosis-like cell death, but not autophagy [107]. Furthermore, no effects on autophagy were observed in vivo and in vitro B16 models using the organic compound *O,O*-diethyl-(S,S)-ethylenediamine-*N,N*′di-2-(3-cyclohexyl) propanoate dihydrochloride, which caused apoptosis, disruption of mitochondrial membrane potential and oxidative stress, leading to decreased tumour growth [108]. Results on the toxicity of copper complexes mononuclear complex 1 and dinuclear complex 2 have shown that susceptibility to these compounds in SK-Mel 05 and SK-Mel 147 cells is related to melanin content and is favoured by UV-B irradiation, which induces melanogenesis, ROS formation, and apoptosis, likely involving autophagic death process [109]. Conversely, apoptosis induction and autophagic flux blockade was detected after the administration of photoactive NADPH analogue NS1 [110]. In particular, NS1 caused cancer cell death by inhibition of NADPH oxidases NOX in A375, SK-Mel 28 and in primary melanoma cells from patients. In addition, NS1 established an early ER stress induced by calcium-dependent redox-sensitive ion channels. These events initiated apoptosis and autophagy, although the autophagic process was incomplete.

### 3.3. Natural Substances

The importance of antioxidant polyphenols from nature in opening up new avenues in skin cancer therapies has been highlighted recently [111]. In this line, several natural molecules, alone or in combination with a well-known pharmacological agent, have simultaneously activated apoptosis and autophagy and induced ROS accumulation in A375 cells. Shikonin, a botanical substance extracted from *Lithospermum erythrorhizon*, exerted antiproliferative action, led to apoptosis and triggered autophagy, upregulating p38 levels. Of interest, autophagy machinery activated by shikonin exerted a protective role against apoptosis and ROS-mediated ER stress and p38 pathways were involved in both apoptosis and autophagy regulations [112]. In the same cell line, sasanquasaponin III (SQS III), a member of SQS class of triterpenoid saponins isolated from theaceous plant, acted as an inhibitory molecule on viability and induced apoptosis and autophagy, both related to ROS accumulation [113]. The powerful anticancer activity of SQS III suggested that ROS are crucial at the crossroad between autophagy and apoptosis. Indeed, SQS III activated autophagy by inhibition of the Akt/mTOR signalling pathway, which promoted apoptosis, and produced an accumulation of intracellular ROS together with alterations of mitochondrial membrane. Graveoline, a bioactive compound isolated from *Ruta graveolent*, induced apoptosis, ROS generation and beclin-1 associated autophagy, although the inhibition of autophagy did not affect cell death nor ROS levels [114]. Using in vivo A375-xenografts, both autophagy and apoptotic cell death appeared to be mediated by ROS production after chalcone flavokawain B treatment [115]. Similarly, a B16-F10 xenograft model was used to test the efficacy of green tea polyphenol analogues JP8 in reducing tumour growth [116]. Indeed, it was observed that JP8 exerts significant anticancer effects reducing tumour progression and it selectively induced ROS leading to ER stress-mediated apoptosis. In another set of experiments, Polygonatum cyrtonema lectin, purified from the rhizomes of *Polygonatum cyrtonema Hua*, a traditional Chinese medicinal herb from Liliaceae, led to the induction of apoptosis and also to autophagy ROS-p38-p53 mediated pathway in A375 cells [117]. The same signalling pathway in A375 cells is activated by other natural compounds including physalin A, a steroidal constituent of Physalis plants, that induced apoptosis via p53-mediated ROS generation and protective autophagy via upregulating the p38-NF-κB pathway [118].

The cytotoxic and pro-apoptotic mechanisms of climacostol, a natural product of the ciliated protozoan *Climacostomum virens*, and its analogues were recently reported in different tumours, including both in vitro and in vivo melanomas (i.e., B16-F1, B16-F10, A373, SK-Mel 5) [119,120,121,122,123]. In particular, climacostol caused a reduction of viability/proliferation of melanoma cells, caused rapidly occurring DNA damage and induced the intrinsic apoptotic pathway involving oxidative-stress-related molecules. Of interest, climacostol potently and selectively impaired autophagy in cells that were committed to die by apoptosis via p53-AMPK axis, although the mTOR pathway unrelated to p53 levels played a role. Thus, in agreement with the promising paradigm of dual targeting of autophagy and apoptosis, data in B16 cells indicated that autophagy and apoptosis might be two separate events in melanoma. They act independently on life/death decisions of the cell and the p53 system is at the molecular crossroad, regulating both the anti-autophagic action of climacostol and its role in the apoptosis induction [122,123]. Other studies conducted in B16-F10 cells confirmed that the ROS-p38-p53 pathway mediates the response to natural compounds, for instance *Marrubium vulgare* (a European medicinal plant) ethanolic extract (MVE) [124]. MVE blocked the cell cycle upregulating p38 and p53 and induced oxidative stress, while antioxidants abrogated its antitumour effect. Furthermore, MVE induced mitochondrial apoptotic pathway and stimulated cytoprotective beclin-1 associated autophagy. Similar results were obtained with citral (3,7-dimethyl-2,6-octadienal), a natural component of essential oils obtained from herbal plants, which induced oxidative stress, autophagy, DNA damage and cell death, also involving p53, NF-κB and Akt signals [125]. In α-melanocyte-stimulating hormone-activated B16-F10 cells, the antioxidant *Penthorum chinense Pursh* ethanol extract reduced LC3 and melanin contents [126]. Recently, antimelogenic effects of ellagic acid, a natural polyphenol produced by hydrolysis of ellagitannins, have been demonstrated in B16-F10 cells. Results also showed that ellagic acid inhibited cell viability and induced autophagy by suppression of PI3K/Akt phosphorylation and mTOR expression [127]. Cytotoxic activity, apoptosis and autophagy induction and high level of ROS production were observed in B16-F10 cells treated with supercritical CO_2_ extract of *Lichen U. barbata* (old man’s beard), which contains usnic acid as the most abundant component [128]. In this line, natto freeze-drying extract and natto water extract, isolated from natto, soybeans fermented by *Bacillus subtilis natto*, were considered to play a critical role in melanoma cell death through ROS adjustment, autophagy regulation and apoptosis promotion [129]. In addition, dimethylacrylshikonin, isolated from the roots of *Onosma paniculata* (Boraginaceae), induced apoptosis, autophagy, ROS generation and loss of mitochondrial membrane potential in WM164 cell line [130].

A protective action against UV-B-induced ROS generation was observed in human primary dermal fibroblast cell line (hDFs) treated with glycyrrhizic acid, a plant-derived triterpenoid saponin glycoside [131]. Results have shown that glycyrrhizic acid protects UV-B-irradiated hDFs from DNA damage and thus from cell death. It acted against UV-B-induced ROS generation and increased the autophagic response, including a downregulation of p62 and Akt, as a protective reaction strategy to reduce photodamage. Similarly, protective effects from UV-B radiation were observed using resveratrol (a phytoalexin from grape) in human keratinocyte HaCaT cells. Resveratrol pre-treatment decreased UV-B-induced ROS while it enhanced apoptosis and autophagy, likely a strategy to avoid malignant transformation of keratinocytes [132]. Similarly, dihydromyricetin, obtained by Rattan tea, induced apoptosis and cytoprotective autophagy on SK-Mel 28 cells [133]. The pathway proposed indicated NF-κB involvement in dihydromyricetin-induced autophagy and a key role of ROS.

Natural substances may also potentiate the effect of chemical drugs. For instance, curcumin, a well-known compound with multiple biological activities, obtained from the turmeric spice *Curcuma longa*, was tested in combination with tamoxifen in A375 and G361 cell lines [134]. The combined treatment increased mitochondria depolarization/ROS levels and induced autophagy and apoptosis. Furthermore, the cooperative therapy of low concentrated curcumin combined with red united blue light irradiation efficiently induced oxidative stress-mediated cell apoptosis, inhibiting A375 cell growth [135]. This combined approach increased cell death and triggered autophagy. Similarly, the anticancer agent icariside II, obtained from *Herba Epimedii*, potentiated the effect of vemurafenib and induced apoptosis and autophagy in BRAF inhibitor-resistant A2058 (intrinsic resistance) and A375 (acquired resistance) cell lines [136]. Of note, icariside II increased mitochondrial ROS levels in resistant melanoma cells with or without vemurafenib, likely preventing BRAF inhibitor resistance via ROS production. Recently, A2058 cells were used to test kalantuboside B, a natural bufadienolide extracted from *Kalanchoe tubiflora* [137]. Results showed that it exerted a potent antiproliferative effect, induced apoptosis and simultaneously enhanced autophagy as a cell survival mechanism. ROS generation was also involved in kalantuboside B action on A2058 cells. In vivo experiments showed the same antitumour effects of kalantuboside B, which promotes apoptosis and cytoprotective autophagy.

From these observations, the induction of autophagy by natural molecules has emerged as a crucial mechanism of action in oxidative stress/apoptosis regulation, however, autophagy impairment was also observed to play a role. For instance, coumarin compound decursinol angelate inhibited cell growth, Atg5, Atg7, and beclin-1 expression, and the conversion of LC3-I to LC3-II in B16-F10 cells [138], thus resulting in inhibition of autophagy. Furthermore, decursinol angelate generated ROS, reduced the mitochondrial membrane potential and induced apoptosis. To note, the use of in vivo models confirmed these indications.

### 3.4. Physical Treatments

Exposure to UV radiation A (UV-A) (315–400 nm), but not UV-B (280–315 nm), upregulated the autophagy adaptor protein p62 in primary normal human epidermal melanocytes and in immortalised mouse progenitor-like melanocytes, as well as in amelanotic A375 cells [139]. It was suggested that pigmentation reduced UV-A-induced p62, as it was observed in darkly pigmented and immortalised melanocytes. Furthermore, p62 upregulation induced ROS, and was observed also in nevus, malignant melanoma and metastatic melanoma, indicating that p62 may act as a crucial factor in melanoma pathogenesis. In A375 cells pre-treated with acridine derivate 9-phenyl acridine and then UV-A-irradiated, apoptosis appeared mediated by mitochondria dysfunction followed by membrane potential decline [140]. In particular, pre-treated cells were more vulnerable to UV-A-induced damages. 9-Phenyl acridine + UV-A treatment induced cell cycle arrest and thus cell death; autophagy and a high level of ROS were also observed. In another study, the role of mitochondrial flavoprotein dihydrolipoyl dehydrogenase protein, which was downregulated by low intensity UV-A, was assessed in MNT1 and A375 cell lines [141]. Results showed that low levels of dihydrolipoyl dehydrogenase induced autophagic cell death and inhibited in vivo tumour growth and proliferation by increasing ROS production and altering energy metabolism.

Photodynamic therapy (PDT) induces ROS and cell death through the activation of a photosensitizer at a specific wavelength. The combination of chemotherapy with PDT has received attention as an alternative approach to treat cancer, including melanoma [142,143]. PDT was shown to increase ROS production and autophagy, also reducing the proliferation of B16-F10 cells [144]. The toxic effects of the cationic zinc (II) phthalocyanine (Pc13) were recently analysed in A375 cells after photodynamic irradiation [145]. Pc13-PDT treatment exerted toxic effects mediated by ROS, also inducing apoptotic cell death and protective autophagy, suggesting that Pc13-PDT treatment increased autophagy that, in turn, inhibited apoptosis. In SK-Mel 3 cells, the combination of zinc phthalocyanine (ZnPc)-PDT and doxorubicin was observed to produce synergistic cytotoxic effects [146]. Indeed, pre-treatment with ZnPc-PDT, at low doses of laser, increased the sensitivity of cells to doxorubicin. No relevant changes of ROS were observed after chemo-PDT treatment but, remarkably, an increase of autophagic flux as well as of apoptosis was ascertained. In some cases, natural substances potentiate the effect of PDT and other promising physical melanoma treatment, such as red united blue light irradiation. Berberine (a critical alkaloid with photochemical properties)-mediated PDT exerted high cytotoxicity and inhibited the growth of A375 and SK-Mel 19 cell lines. Treatment induced apoptosis, reduced mTOR and Akt proteins and increased LC3II/I, also activating ER stress and ROS [147]. In this respect, the cooperative therapy of low concentrated curcumin combined with red united blue light irradiation efficiently induced oxidative stress-mediated cell apoptosis, inhibiting A375 cell growth [135]. This combined approach increased cell death and triggered autophagy.

## 4. Conclusions: Current Melanoma Therapies and Outlook on RONS/Autophagy-Based Strategies

Early diagnosis of melanoma is crucial. Indeed, if the tumour is treated when is still confined to the epidermis, the prognosis is excellent and the 10-year survival rate is estimated to be about 90–100%. On the contrary, if melanoma is diagnosed at a more advanced stage, the 5-year survival rate drops to 10–15% [148]. Surgical excision is the gold standard for early-stage melanoma and it may be curative in most cases. Until about 10 years ago, late-stage disease patients were difficult to treat due to presence of metastasis, often refractory to most treatments available (classic chemotherapeutic drugs and cytokine-based immunotherapy) at that time.

Since 2011, the approval of several drugs (molecular targeted agents and immune checkpoint inhibitors) for the treatment of metastatic melanoma has increased the chance of survival for patients with advanced disease. The first targeted therapies approved for the treatment of metastatic melanoma reported as effective in delaying the course of the disease (improvements in median progression-free survival and median overall survival) were vemurafenib and dabrafenib. They are both BRAF inhibitors that work on the mutated forms of the protein (V600E for vemurafenib and V600E and V600K for dabrafenib), occurring in almost all patients with BRAF-mutant melanoma [149]. More recently, the USA Food and Drug Administration approved another BRAF inhibitor, encorafenib [150]. However, resistance to this kind of treatment often occurs because of the reactivation of the MAPK pathway and/or other mutations, and leads to disease progression [151]. To overcome this issue, preclinical and clinical trials were carried out combining BRAF inhibitors with small molecule inhibitors that target other molecular players of the MAPK pathway such as MEK [152]. The results of a study evaluating the combination of dabrafenib plus trametinib, a MEK inhibitor, in patients who have unresectable or metastatic melanoma with a BRAFV600E or V600K mutation, demonstrated the long-term benefit in approximately one third of the patients [152]. However, the course of metastatic melanoma treatment has changed markedly since the introduction of the immune checkpoint inhibitors designed against either cytotoxic T-lymphocyte-associated antigen 4 (CTLA-4) or Programmed death receptor-1 (PD-1), both negative regulators of T-cell immune function [153]. Ipilimumab, the inhibitor of CTLA-4, and nivolumab and pembrolizumab, both PD-1 inhibitors, were approved originally for the treatment of advanced melanoma and resected disease as monotherapy [154,155]. In 2016 the European Medicines Agency approved the combination of ipilimumab and nivolumab, based on the phase III clinical trial CheckMate 067, in which the combination was compared with the two monotherapy regimes [155,156]. The combination therapy group showed significantly higher progression-free survival and overall survival than both nivolumab and ipilimumab groups. However, the incidence of adverse reactions was also higher in the combination therapy group and the current guidelines recommend this strategy in patients accepting the increased toxicity [157].

Unfortunately, although the treatment of metastatic melanoma has greatly improved in recent years, a significant subset of patients do not show a long-lasting response to these treatments. Therefore, unravelling new processes and their interconnections underlying the initiation/progression of the disease may help to identify new reliable pharmacological approaches for the development of more effective and durable therapies. There are controversial (even opposite) results about the use of potential antioxidant molecules or autophagy modulators in melanoma therapy [63,64,72]. However, although evidence supporting RONS and autophagy as dual targets is promising, mechanistic evaluation of their cross-talk and relationship is complex.

**Figure 2:**
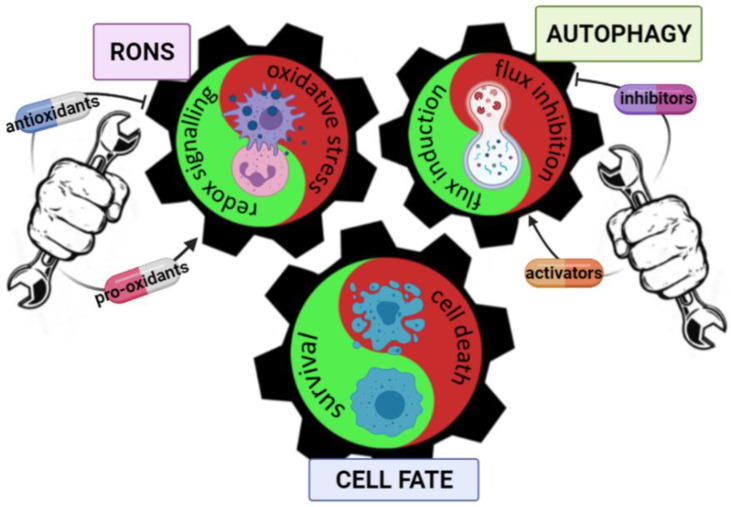
The mechanistic cross-talk between oxidative stress and autophagy in melanoma cell fate.

As previously suggested by Hambright and Ghosh [31], one particular challenge is first recognizing the significance of targeting two mechanisms which may be simultaneously pro-survival and pro-apoptotic for cells (Figure 2). In other words, RONS may act both as stimulatory and detrimental factors for melanoma viability, as does autophagy. RONS and autophagy machineries seem to have a hormetic effect on melanoma cell fate. Differential roles on cell response rely on threshold levels of oxidative stress and autophagy turnover (which can influence each other). In most cases, melanoma fate switches to death as RONS increases beyond the scavenging potential of the cell. As RONS are induced, the catabolic process response is the activation of the autophagosome turnover in an attempt to clear the cell of oxidized/damaged contents, but the blockade/inhibition of the pro-survival autophagic mechanisms may also occur. However, opposite molecular dynamics can also arise in the RONS/autophagy balance. We note that starting from preclinical studies it is essential to evaluate in depth the usefulness of pro-oxidants/antioxidants and the parallel activation/inhibition of autophagy in each distinct case. The complication of the picture depends on multiple factors, for instance in vitro vs in vivo models, melanoma cell types, combination approaches, action mechanism of the compound and its pharmacology, treatment outcomes, proliferation/survival curves, tumour burden and progression-free survival. In conclusion, the development of successful therapies in melanoma depends on a more complete understanding of the cross-talk between oxidative stress and autophagy as well as its pharmacological targeting. These challenges deserve consideration to better define drug response for stratified medicine, also being useful to find valuable and innovative markers/targets in patients.
